# Optimized perioperative management (fast-track, ERAS) to enhance postoperative recovery in elective colorectal surgery

**DOI:** 10.3205/dgkh000413

**Published:** 2022-06-23

**Authors:** Wolfgang Schwenk

**Affiliations:** 1 GOPOM GmbH, Gesellschaft für Optimiertes PeriOperatives Management, Düsseldorf, Germany

**Keywords:** optimized perioperative management, enhanced recovery after surgery, ERAS, fast-track surgery, enhanced recovery protocols, morbidity, perioperative medicine

## Abstract

**Aim:**

This manuscript provides information on the history, principles, and clinical results of Fast-track or ERAS concepts to optimize perioperative management (OPM).

**Methods:**

With the focus on elective colorectal surgery description of the OPM concept and its elements for with special attention to the prevention of infectious complications and clinical results compared to traditional care will be given using recent systematic literature reviews. Additionally, clinical results for other major abdominal procedures are given.

**Results:**

An optimized perioperative management protocol for elective colorectal resections will currently consist of 25 perioperative elements. These elements include the time from before hospital admission (patient education, screening, and treatment of possible risk factors like anemia, malnutrition, cessation of nicotine or alcohol abuse, optimization of concurrent systemic disease, physical prehabilitation, carbohydrate loading, adequate bowel preparation) to the preoperative period (shortened fasting, non-sedative premedication, prophylaxis of PONV and thromboembolic complications), intraoperative measures (systemic antibiotic prophylaxis, standardized anesthesia, normothermia and normovolemia, minimally invasive surgery, avoidance of drains and tubes) as well as postoperative actions (early oral feeding, enforced mobilization, early removal of a urinary catheter, stimulation of intestinal propulsion, control of hyperglycemia). Most of these elements are based on high-level evidence and will also have effects on the incidence of postoperative infectious complications.

**Conclusion:**

Optimized perioperative management should be mandatory for elective surgery today as it enhances postoperative patient recovery, reduces morbidity and infectious complications.

## Zusammenfassung

**Zielsetzung:**

Dieses Manuskript informiert über die Geschichte, Prinzipien und klinischen Ergebnisse von Fast-Track- oder ERAS-Konzepten zur Optimierung des perioperativen Managements (OPM).

**Methoden:**

Mit dem Schwerpunkt elektiver kolorektaler Operationen werden das OPM-Konzept und seine Elemente mit besonderem Augenmerk auf die Vermeidung infektiöser Komplikationen und die klinischen Ergebnisse im Vergleich zur traditionellen Versorgung anhand einer aktuellen Literaturübersicht beschrieben. Zusätzlich werden klinische Ergebnisse für andere größere abdominale Eingriffe angegeben.

**Ergebnisse:**

Ein Protokoll zum optimierten perioperativen Management für elektive kolorektale Resektionen besteht derzeit aus 24 perioperativen Elementen. Diese Elemente umfassen von der Zeit vor der Krankenhausaufnahme (Patientenaufklärung, Screening und Behandlung möglicher Risikofaktoren wie Anämie, Mangelernährung, Beendigung des Nikotin- oder Alkoholmissbrauchs, Optimierung gleichzeitiger systemischer Erkrankungen, körperliche Prähabilitation, Kohlenhydratbelastung, adäquate Darmvorbereitung) über die unmittelbare präoperative Phase (verkürztes Fasten, nicht-sedative Prämedikation, Prophylaxe von PONV und thromboembolischen Komplikationen), die intraoperativen Maßnahmen (systemische Antibiotikaprophylaxe, standardisierte Anästhesie, Normothermie und Normovolämie, minimalinvasive Chirurgie, Vermeidung von Drainagen und Schläuchen) und die postoperativen Elementen (frühzeitige orale Ernährung, forcierte Mobilisierung, frühzeitige Entfernung eines Harnkatheters, Stimulation des Darmtransports, Kontrolle der Hyperglykämie). Die meisten dieser Elemente beruhen auf wissenschaftlichen Studien mit hohem Evidenzlevel und wirken sich auch auf das Auftreten postoperativer infektiöser Komplikationen aus.

**Schlussfolgerung:**

Das optimierte perioperative Management sollte heute bei elektiven in allen Bereichen der Chirurgie obligatorisch sein, da es die postoperative Genesung des Patienten fördert, die Morbidität senkt und dabei auch die Häufigkeit infektiöser Komplikationen verringert.

## Introduction

Evidence-based, multimodal, and evidence-based concepts to optimize the outcome of patients who undergo surgery have been published for almost three decades now [1]. “Fast-track”, “ERAS” (short for “enhanced recovery after surgery”), optimized track, or rapid recovery are different synonyms describing these perioperative treatment protocols. Within the last decade, terms like ERAS or Fast-track have also become trademarks for companies that provide hospitals with a structured program to implement these concepts into the clinical routine. Therefore, it seems prudent to use the neutral term “optimized perioperative management” (OPM) to describe these perioperative protocols in this manuscript.

It should be noted here that the term ERAS^®^ is under worldwide legal protection of the ERAS^®^-society and the ERAS Implementation Program (EIP) is run by Encare, Sweden. Fast-track is part of the term Go FAST-TRACK^®^ an implementation program offered by the German GOPOM GmbH, owned by the author of this manuscript.

In 1995, the Danish surgeon Henrik Kehlet and his team published a series of 8 elderly patients who underwent laparoscopic resection of colonic cancer and were treated by a special perioperative program [1]. Their protocol included patient counseling and education, optimal pain treatment, normothermia, and euvolemia as well as early postoperative oral feeding and enforced mobilization on the day of surgery. Patients were discharged without morbidity on day two after surgery. In 2005 the ERAS-study group, founded by Olle Ljungqvist (Sweden), Ken Fearon (Scotland), Arne Revhaug (Norway), Martin von Meyenfeldt and Cornelius deJong (Netherlands), as well as Henrik Kehlet, published the first consensus protocol on optimized perioperative management for patients undergoing colonic surgery [2]. In 2010 the ERAS^®^ society was founded and has since then published several consensus protocols on optimized perioperative management in all surgical specialties (https://erassociety.org/guidelines/, last accessed February 28^th^ 2022). Within these 25 years, OPM protocols have spread from elective colonic resections to other procedures in abdominal, thoracic, vascular, orthopedic, and cardiac surgery, as well as gynecology, urology, ENT, and oral and maxillofacial surgery.

In general, OPM protocols are evidence-based, multimodal, and interprofessional recommendations for physicians, nursing personnel, and other medical staff involved in perioperative management. These protocols include up to 20 or more different elements and are specific for certain procedures or groups of procedures. Although OPM has been studied extensively in all kinds of surgical procedures, the protocol for elective colorectal surgery [3] remains to be the best-evaluated guideline and shall therefore be described in further detail.

## Method

The PubMed database of the US National Library of Medicine was searched using the following algorithm: 

("fast-track"[Title] AND "systematic review*"[Title]) OR ("eras"[Title] AND "systematic review*"[Title]) OR ("enhanced recovery"[Title] AND "systematic review*"[Title]) OR ("enhanced recovery"[Title] AND "meta analysis*"[Title]) OR ("eras"[Title] AND "meta analysis*"[Title]) OR ("fast-track"[Title] AND "meta analysis*"[Title]).

Titles and abstracts of the retrieved literatures were checked to see if they met the following criteria:

- systematic reviews with meta-analysis of studies comparing fast-track/ERAS and traditional perioperative care,
- data on complications, lethality, functional recovery, postoperative hospital length of stay, and readmission rate after discharge from inpatient care,
- surgical procedures from general, and visceral surgery,
- publication in English or German.

Excluded were

- narrative literature reviews or systematic reviews with descriptive data analysis without statistical meta-analysis,
- comparisons of different ERAS protocols,
- Reviews assessing individual elements of the fast-track/ERAS concept (e.g., surgical technique or analgesia procedures) in the context of traditional perioperative treatment structures or established enhanced recovery protocols,
- Reviews of fast-track/ERAS management in gynecologic, urologic, ENT, or neurosurgical operations.

From the publications, those with the most current literature review were further considered. Ideally, meta-analyses of randomized controlled trials (RCTs) were considered in isolation. Data describing the postoperative course such as morbidity (general and local complications, severity of complication), functional recovery parameters (e.g., time interval to first bowel movement) were summarized in when available. 

## Results

### Optimized perioperative management in elective colorectal resections

The OPM protocol for patients undergoing elective colorectal surgery describes 24 different elements which should be adhered to from before admission of the patient to the hospital to the preoperative, intraoperative, and postoperative period of treatment [3], [4]. These elements are shown in Table 1 (Tab. 1).

#### Preadmission elements of OPM

Optimized perioperative management protocols start well before admission of the patient to the hospital. Beyond proper diagnosis of the primary disease that leads to surgery, the preadmission period is essential to inform and educate the patient, diagnose, and treat concomitant diseases as well as malnutrition or anemia and allow for the preoperative cessation of alcohol and nicotine abuse. 

#### Patient education

In traditional perioperative treatment patient counseling focuses on surgery and anesthesia. Due to legal requirements, patients must sign an informed consent which includes rigorous education on risks and complications of the operative procedure and the anesthesia provided. It has been shown that pre-operative education can increase patients’ knowledge and satisfaction and may reduce anxiety levels [5]. However, clinicians’ skill to deliver reassuring information varies considerably, and time and resource pressures may act as a severe barrier to extensive patient counseling and education. Within an OPM pathway, extensive patient education and information face-to-face and by written material decreased postoperative analgetic dose, time to first bowel movement, and hospital stay after colorectal surgery significantly. At the same time, oral fluid intake and time out of bed were increased in patients that underwent extensive education in this randomized controlled trial (RCT) [6]. Extensive patient education and counseling especially concerning his active part in postoperative recovery are strongly recommended! 

#### Optimization

Medical optimization of concomitant diseases is based on adequate patient assessment. Preoperative evaluation and treatment of underlying cardiovascular and pulmonary disease have been targeted by several guidelines [7], [8] and expert recommendations [9]. However, clinical trials have shown, that adequate diagnosis of cardiovascular disease is complex, and most patients will be either underdiagnosed or overdiagnosed even by experienced physicians [10], [11]. Except for thorough anamnesis and physical examination, no further diagnostic tests may be necessary at all in healthy patients with adequate functional capacity, extensive testing under cardiological guidance may be unavoidable in very few high-risk patients. Pure “clinical judgment” is misguiding many patients and should be replaced by a more structured approach to risk assessment using checklists or risk calculators [12]. 

#### Prehabilitation

Prehabilitation combines all efforts to optimize the patient before surgery either by psychological aid, physical training, or cessation of nicotine or alcohol. 

#### Psychological treatment

Psychological support may be helpful, especially in cancer patients, and will encourage the patients to be more active in the process of postoperative recovery. However, evidence of a high level concerning the influence of psychological support on postoperative recovery and morbidity of patients was not available to the author of this manuscript. Psychological support may aid the patient in alcohol and nicotine cessation (see below).

#### Alcohol and nicotine cessation

Cochrane reviews on preoperative alcohol cessation have shown a significant decrease of postoperative complications from 54% to 33%, although 121 patients [13]. Slightly larger patient numbers were recruited in RCT included in a systematic review on smoking cessation before surgery [14]. Only 5.6% of 232 Patients who quit smoking 4–6 weeks before surgery experienced a postoperative complication, while morbidity was observed in 13.0% of 184 patients who continued to smoke. Therefore nicotine- and alcohol cessation is strongly suggested 4–6 weeks before surgery. 

#### Physical training

Preoperative physical training may improve cardiopulmonary function and may therefore help to avoid postoperative complications. However, data on physical prehabilitation before abdominal surgery is inconsistent. A systematic review on RCTs on multimodal prehabilitation before abdominal cancer operations showed a significant increase in cardiopulmonary function, measured by an increased VO2max, and better results in the 6-minute-walking-test [15]. Postoperative length of hospital stay was reduced by 3.7 (95% KI 0.92; 6.4) d, but postoperative morbidity was not significantly different (39.3% vs. 46.5%) in this population. A recent small RCT [16] including high-risk patients showed significantly lower morbidity after elective colorectal surgery in 28 patients who underwent a 3-week personalized exercise program (42.9%) compared to 29 patients without training (72.4%). A large Scandinavian RCT [17] recruited 762 patients with lower risk (ASA II-IV: 19%) to 2 weeks of medium intensity aerobic labor (30 min per day) and inspiratory muscle training (30x2 breaths per day) (n=31/) or no physical training (n=351). Postoperative complications were not different between both groups as were readmission rate and self-assessed physical recovery. While not all patients may benefit from physical training, negative side effects are rare, and moderate aerobic training is recommended especially in high-risk patients before colorectal surgery. 

#### Treatment of anemia

Female patients with hemoglobin <12 g% and males with hemoglobin <13 g% are considered anemic. The prevalence of anemia is as high as 10–48% in surgical patients, especially in those undergoing vascular, cardiac, gynecological, orthopedic, urological, or colorectal surgery. Mortality is increased from 0.95% to 4.6% in patients who are anemic and undergo non-cardiac surgery, while the of perioperative blood transfusions significantly increases from 3.1% to 15.0% [18]. Diagnosis of anemia is simple but according to the S3-guideline on preoperative anemia, adequate treatment requires more extensive laboratory work to discriminate between the most common cause of anemia in patients undergoing colorectal resection, which is iron deficiency, and more rare causes of anemia in this population like Vitamin B_12_ or folic acid deficiency, renal anemia, and other rare conditions. The S3-guideline does not recommend “blind” treatment with oral or intravenous iron without proof of iron deficiency is the cause of anemia and no recommendations were given concerning the preoperative treatment of anemia with oral or i.v. iron. A recent systematic review of RCTs [19] showed a significant risk reduction for transfusion from 40% to 33% when anemic patients were treated with intravenous iron compared to a placebo. At the same time, hemoglobin was increased by 0.7 g% before and 0.6 g% 4 weeks after surgery in patients who received i.v.-iron. It is recommended to diagnose patients with preoperative anemia, postpone surgery and treat anemia according to the diagnostic findings.

#### Screening and treatment of malnutrition

Untreated malnutrition increases postoperative morbidity and mortality in almost any kind of surgery. Therefore, the S3-guideline from 2013 [20] (currently under revision) recommended that screening for malnutrition should be performed in any patient before major surgery, which of course includes colorectal resection. Simple questionnaires like the Nutritional Risk Score 2002 [21] can be used to determine patients at risk for malnutrition. If malnutrition is diagnosed, patients who can eat normally (as most elective colorectal patients will) should be treated with additive protein drinks for 7–10 d before surgery. Enteral or even parenteral feeding is rarely needed in colorectal patients, but, if necessary, should also be given for the above-mentioned amount of time. 

All preadmission OPM elements, with levels of evidence and grade of recommendation, are given in Table 2 (Tab. 2). 

### Preoperative elements of OPM

If possible, patients should be admitted to the hospital on the day of surgery. Therefore, the OPM elements described in this part of the manuscript should be provided to the patient before admission to the hospital. As it may be unavoidable to admit some patients to the hospital the day before surgery, the elements within 24 hours before surgery are combined as preoperative elements.

#### PONV-prophylaxis

Postoperative nausea and vomiting is a common syndrome that has been very well studied in the last decades. The following factors identify patients at risk for PONV: female sex, younger age, non-smoker, and history of travel sickness or PONV, surgery lasting >1 h, opioid medication during or after surgery, and certain types of surgery. All patients should be assessed for their risk and prophylaxis should be administered accordingly. In 2020 a Cochrane Review [22] collected data from 585 RCT including 97,516 patients that investigated medical PONV prophylaxis. Currently, several drugs are available: 5-HT3-antagonists, D2-receptor antagonists, NK1-receptor-antagonists, corticosteroids, antihistaminic and anticholinergic drugs. Other measures to mitigate the risk are: avoidance of volatile anesthetics, performing regional anesthesia, and/or using total intravenous anesthesia with as few opioids as possible. According to the American guideline from 2020 [23] patients with 1–2 risk factors should receive two drugs, most likely a 5-HT3-antagonist and dexamethasone, patients with 3–4 risk factors should receive 3–4 of the drugs mentioned above. It is still debatable if a patient with 0–1 risk factor should also receive dexamethasone because side effects are very rare and additional positive effects of dexamethasone may include decreased postoperative pain and better pulmonary function.

#### Adequate bowel preparation and oral antibiotics

For more than 5 decades mechanical bowel preparation (MBP) using saline solutions, laxatives, and/or polyethyleneglycol solutions has been a standard procedure before colorectal surgery. MBP was administered to “clean” the bowel and decrease postoperative SSI and namely anastomotic leakages. Furthermore, oral antibiotics given from 1–3 d before surgery were also a traditional way to prepare the bowel before colorectal resection (not to be confused with „selective bowel decontamination“ which usually includes antimycotics and may last from up to 5 d before to 7 d after surgery). Several meta-analyses of RCTs were not able to show any advantage of MBP in terms of SSI and especially anastomotic leakage [24], [25], while oral antibiotics (also “forgotten” in the last decade of the 20^th^ century) were shown to be effective in RCTs [26]. In recent years, several publications from the register of the American College of Surgeons revived the interest in MBP and oral antibiotics [27], [28], [29]. 

In a systematic review of 23 RCTs and 13 observational studies including 6,277 and 12,663 patients respectively [30] no difference was found in anastomotic leak rate when MBP was compared to no bowel preparation (3.5% vs. 4.6%) or compared to a small enema (4.5% vs. 5.4%). Furthermore, there were no significant differences in the incidence of SSI, reoperation, mortality, or length of stay between these groups. Therefore, MBP alone cannot be recommended.

In 2019 another systematic review [31] including 28 RCT again found no significant difference in anastomotic leak rates in patients treated either by MPB alone or by MBP + *oral antibiotics*. However, the incidence of SSI was significantly reduced from 14.5% to 8.2% when MBP + oral antibiotics were given. Other outcomes did not differ between these groups. 

A network meta-analysis of RCT published in 2018 [32] including 38 RCT with 8,458 patients did find a significantly reduced risk of wound infection when MBP + oral antibiotics were used, compared to MBP alone or no bowel preparation. Again, there were no differences in anastomotic leak rate or other adverse events. A recently published RCT [33] including 582 patients randomized to oral antibiotics (n=282) no oral antibiotics (n=282) but did not give any MBP found that SSI was significantly less common in patients who received oral antibiotics (19% vs. 28%).

In 2022, several large multicenter RCTs are conducted worldwide to further enhance our understanding of the value of MBP and oral antibiotics in elective colorectal surgery. Today, the application of *MBP* alone is not recommended, but it can be given if combined with oral antibiotics [3].

#### Carbohydrate drinks

Preoperative fasting may contribute considerably to patient discomfort and normovolemia is important to maintain/restore patient autonomy as soon as possible after surgery. Furthermore, filling glycogen stores in muscle and liver before surgery may have positive effects on postoperative metabolism. Therefore, the administration of carbohydrate drinks on the day before surgery and the morning of the operation serves several purposes. According to a recent meta-analysis of 57 RCTs with 5,606 patients [34], preoperative oral carbohydrates improved the postoperative discomfort in terms of dryness of mouth, thirst, hunger, pain, and vomiting statistically significant when compared to a control group. Postoperative insulin resistance was improved in the carbohydrate patients and even the length of stay was shortened by 0.39 d. In one RCT 662 patients undergoing elective abdominal surgery were randomized to carbohydrate drinks (n=331) or water (n=331) before surgery [35]. Significantly fewer patients from the carbohydrate group (24,2%) compared to the water group (57.4%) showed blood sugar levels above 140 mg/dl postoperatively and only 2.4% of the carbohydrate group received insulin injections compared to 16.0% of the water group. Incidence of postoperative complications (28.1 vs. 28.4%) and infections (16.3% vs. 16.0%) were not different between groups. Although recent guidelines allow patients solid food up to 6 hours and clear (non-fat) liquids up to 2 hours before surgery, some surveys show, that many of all patients are kept on a “nil-per-mouth”-strategy for a much longer time [36], [37], [38]. OPM protocols encourage patients to drink 400 cc of a 12% carbohydrate solution the afternoon before surgery and a further 200 cc carbohydrate solution 2 hours before surgery.

#### Non-sedative premedication

Sedative premedication before surgery may lead to prolonged awakening times after general anesthesia and may contribute to postoperative delirium in elderly patients. RCTs have shown that premedication with long-acting sedatives like Lorazepam will not decrease anxiety but lengthen the time to extubation as well as the cognitive recovery 40 minutes after surgery [39]. Also, sedative premedication was associated with “oversedation” while feeling anxious or not feeling anxious before surgery was not correlated to whether a sedative was given or not [40]. OPM protocols recommend not to use sedative premedication at all or at least only in very few selected patients.

#### Intravenous (i. v.) antibiotic prophylaxis

Intravenous antibiotic prophylaxis is a cornerstone to prevent postoperative infectious complications after elective colorectal surgery, namely SSI. Typically, a 2^nd^ or 3^rd^ generation cephalosporine is combined with metronidazole and given as a “single-shot” within 60 minutes before skin incision. This dose is repeated after 3 hours if surgery lasts longer than that or if severe bleeding occurs. In 2017, a systematic review and meta-analysis of 9 observational trials showed that the risk of SSI is significantly increased if i. v. antibiotics are administered after skin incision or more than 120 minutes before the beginning of surgery [41]. There was no significant difference if antibiotics were given less than 30 minutes before surgery or between 30 and 60 minutes before skin incision. In the clinical routine, administration of i. v. antibiotics is performed by the anesthetist before or during induction of anesthesia. Also, the administration of antibiotic prophylaxis is one of the key questions in the WHO safety checklist [42]. If team time out before incision reveals that i. v. antibiotics have not been given, the administration should be performed immediately, and skin incision should be delayed for 5–10 minutes. Antibiotic prophylaxis should not be continued after surgery, as another systematic review has proven, that there is no additional benefit of postoperative continuation of antibiotic prophylaxis [43].

All preoperative OPM elements required are listed in Table 3 (Tab. 3).

### Intraoperative elements of OPM

#### Standardized anesthesia

The international consensus conference has given some advice on how to conduct anesthesia for elective colorectal resections [3]. However, a detailed recommendation concerning anesthesia is not provided and this would certainly exceed the scope of the recommendations and this manuscript. However, the following principles of anesthesia should be adhered to.

Long-acting sedatives and opioids should be avoided. Propofol is suggested for induction and total intravenous anesthesia (TIVA) with short-acting opioids (i. e. fentanyl, alfentanil, sufentanil) is recommended but modern volatile anesthetics (sevoflurane, desflurane) may also be used. In patients with a high risk for PONV, TIVA seems to be preferable to volatile anesthetics. Cerebral function monitoring using the bi-spectral index (BIS) should be considered. Monitoring of muscle relaxation is advised to allow for low abdominal wall tension in laparoscopic surgery and avoid inadequate reversal of neuromuscular block at extubation.

#### Intraoperative fluid and volume therapy

Although intravenous fluid and volume therapy is a fundamental technique of anesthesia and intensive care, until today the “perfect” amount of i. v. fluids for a given operation is not clearly defined. Therefore, OPM recommendations are not very precise [3]. It is recommended not to use NaCl 0.9% solution for fluid therapy but rather balanced electrolyte solutions. The general goal of fluid management is to avoid “overloading” as well as “dehydrating” the patient. In general, a “near-zero” fluid balance should be aimed at. A gain of bodyweight >2.5 kg is associated with increased morbidity and should be strictly avoided. Therefore, in an uneventful operation, intraoperative fluids should be limited to 1.5–2.0 l and vasoactive drugs should be used to maintain adequate circulation if necessary. The amount of fluids given should not be guided by urine output. In high-risk patients, invasive hemodynamic monitoring may be helpful to guide a “goal-directed” approach to fluid therapy. 

#### Normothermia

The maintenance of a body temperature between 36.0° and 37.5° Celsius is extremely important to avoid postoperative complications. As a rule of thumb, per 1.0° Celsius of hypothermia, postoperative complications including general complications as well as wound healing impairment and other infectious complications, will increase by 10%. As described in the German S3-Guideline from 2019 [44], the following measures should be initiated to achieve normothermia even during prolonged surgery: active preoperative warming using warm air blankets or warm mats, active warming during surgery, preferable with warm air covers and warming mats, warm i. v. fluids (effective if >500 cc per hour), room temperature >21° Celsius and warm irrigation fluids (38°–40°C). Postoperative shivering is not only uncomfortable for the patient but also increases peripheral oxygen demand considerably putting the cardiovascular system under stress. If postoperative shivering occurs, it should be treated by active warming and the “off-label” use of pethidine and clonidine.

#### Operative technique

Good operative technique with attention to avascular planes of tissue is a prerequisite to achieving a good postoperative outcome for the patient. Whenever feasible, minimally invasive surgery should be used because it results in less pain, better pulmonary function, shorter duration of postoperative ileus, and thereby improves patient recovery [45]. No effect of laparoscopic surgery on general complications (i. e. cardiovascular, pulmonary events) has been demonstrated so far, but local complications (i. e. SSI, fascial dehiscence) are less common if minimally invasive surgery is compared to open surgery [46], [47].

#### Avoid tubes and drains

It is well known since 2011 [48] that avoidance or early postoperative removal of a *nasogastric* tube after colorectal surgery will result in a significantly lesser incidence of airway infections (2.6 vs. 6.9%) and earlier tolerance of oral feeding (2.6 vs. 5.0 d). Prolonged postoperative use of a nasogastric tube will not decrease nausea (19.8 vs. 24.4%) although vomiting may be less common (10.9 vs. 31.1%). Nasogastric tubes should not be used or removed at the end of surgery.

Traditionally intraabdominal or subcutaneous drains are used to detect postoperative complications or even to avoid them. In 2016 a systematic review of 11 RCT showed that intraabdominal drains will not affect the incidence of leakage in intraperitoneal (3.5 vs. 3.2%) and extraperitoneal (12.3 vs. 12.4%) anastomoses [49]. In 2020, another meta-analysis of RCTs supported these findings but found a significantly increased risk for postoperative ileus when drains were used (9.9 vs. 6.9%) [50].

#### Intraoperative multimodal analgesia

The main purpose of intraoperative multimodal analgesia is to avoid or at least reduce opioids because opioid-related side effects like nausea, vomiting, prolonged sedation, or gastrointestinal paresis will alter postoperative recovery. Regional analgesic techniques should be utilized if possible. Thoracic epidural analgesia (thEDA) with a local anesthetic and an opioid has been the preferred type of regional analgesia in open as well as laparoscopic surgery for years. Lately, however, side effects of EDA have come to attention, namely hypotension and impaired urinary bladder function [51]. While thEDA is still the technique of choice in open colorectal surgery, other regional techniques are recommended in laparoscopic surgery. The consensus guideline recommends spinal analgesia with a local anesthetic/opioid mixture or bilateral transversus abdominis plane blocks (TAP-block) with a local anesthetic as the first choice in these cases [3]. If these techniques are not possible, continuous intravenous Lidocaine may be used. However, side effects of either technique must be considered: motor block is inevitable in spinal analgesia and will not allow for mobilization of the patient and/or removal of the urinary catheter for some hours postoperatively. i. v. Lidocaine requires continuous monitoring of heart rate due to its (anti)arrhythmic properties. 

All intraoperative elements of OPM are given in Table 4 (Tab. 4).

### Postoperative elements of OPM

#### Urinary catheter

Traditionally, urinary catheters have been used for 2–5 d after surgery. Especially in rectal surgery, urinary catheters remained in place for more than 3 d, because bladder dysfunction was supposed to be more common due to irritation of autonomous nerves. This practice was subject to criticism because urinary catheters are not only uncomfortable for patients but are also associated with urinary tract infections and injury to the urethra (especially in men). A meta-analysis of 4 RCTs including 409 patients evaluated the early removal of urinary catheters after rectal surgery [52]. Patients with early removal of the urinary catheter were significantly more prone to re-catheterization (18.8 vs. 9.4%), but urinary tract infections occurred in 21% of the patients with late removal, while it was only detected in 9.7% when the catheter was removed early (p<0.05). Similar results were obtained by a meta-analysis including pelvic colorectal surgery [53]. OPM protocols recommend early removal of the urinary catheter, either at the end of surgery or within 24 hours later.

#### Early oral feeding

Early oral feeding is usually avoided in traditional care because nausea and vomiting, aspiration, and impaired anastomotic healing were feared. Usually, the patient stayed on clear liquids for at least 24–48 hours before feeding was stepped up to soup, smashed food, and finally a regular diet. The influence of early or delayed postoperative oral feeding has been evaluated in several studies. 2018 a meta-analysis of 9 RCTs including 879 patients evaluated the effect of early versus delayed oral feeding on anastomotic leakage after elective lower intestinal surgery [54]. Anastomotic leakage was significantly more common in patients with delayed (4.5%) versus patients with early feeding (1.4%). Furthermore, postoperative morbidity was significantly lower in patients who received early oral feeding (19.5 vs. 26.0%).

##### Early oral protein-containing diet

Protein content of oral food may have a relevant influence on postoperative healing and some authors have voted for protein-containing diets for early oral feeding. Another systematic review of RCT published in 2021 [55] showed that early feeding with protein-containing food compared to late traditional feeding did not only reduce postoperative SSI (4.2 vs. 10.7%) but lowered postoperative morbidity (4.2 vs. 1.2%) and postoperative length of hospital stay (by 2.1 d). OPM suggests starting postoperative oral feeding on the day of surgery and using protein-enriched drinks as a supplement to regular food at least for 3–7 d after surgery.

#### Prevention of postoperative ileus

Under traditional therapy postoperative ileus occurs in 10% of all patients undergoing colorectal surgery, leading to bloating, abdominal pain, nausea, and vomiting, preventing oral intake, and causing electrolyte disturbances and other complications like aspiration. Risk factors associated with postoperative ileus are male sex, advanced age, concomitant cardiac disease, open surgery, and creation of a stoma [56]. Numerous drugs have been tested to prevent postoperative ileus and the following measures have been proven to be effective in RCTs: thoracic epidural analgesia, minimally invasive surgery, continuous intravenous lidocaine. Peripheral u-receptor antagonists have also been shown to be effective but are not available in Germany so far. While erythromycin or soluble contrast media did not show significant improvements, non-steroidal anti-inflammatory drugs are still under investigation because of their positive influence on inter-enteric inflammatory reaction after surgery. High amounts of i. v. fluids and salt during surgery will also lead to a higher incidence and more severe cases of postoperative ileus. Several non-medical treatments have also been shown to be effective in preventing postoperative ileus or shortening its course: coffee, chewing gum acupuncture, and Daikenchuto (a traditional Japanese herbal medicine). Early oral feeding has a clinically relevant effect on postoperative ileus. To prevent postoperative ileus, it is recommended to avoid opioids and fluid overload, use minimally invasive surgery or regional anesthesia, feed patients early, serve coffee and ask the patient to use chewing gum 3 times 15 minutes per day. The regular use of NSAIDs is currently under debate because their impact on prostaglandin metabolism may impact wound healing and favor anastomotic leakage. 

#### Control of blood sugar

Posttraumatic metabolism and insulin resistance cause postoperative hyperglycemia. Persistent or recurrent hyperglycemia may alter postoperative wound healing and can be excessive even in patients who were not diabetic before surgery. A recent observational study found an association between postoperative hyperglycemia and anastomotic leakage in previously non-diabetic patients [57]. 15% of non-diabetic patients with blood sugar peaks above 200 mg% experienced anastomotic leakage after colorectal resection, while this was less common in patients with peaks between 126–200 (12%), 101–125 mg% (5%), and less than 100 mg% (3%). Preoperative metabolic conditioning by carbohydrate drinks will decrease the incidence of postoperative hyperglycemia (see above) but blood sugar should be monitored regularly after surgery and patients should be treated accordingly if hyperglycemia is detected.

#### Early mobilization

Early mobilization on the day of surgery is a fundamental element of OPM protocols. However, scientific evidence on the positive effects of early (and sometimes enforced) postoperative mobilization is rarely found. On the other hand, it is well known since 1999 that bedrest is a harmful treatment. At that time review of RCTs [58] demonstrated that prophylactic bedrest never resulted in a positive result, was harmful in 8 studies, and did not improve the outcome in 16 studies. When bed rest was a primary treatment, it did again not achieve any positive results, worsened the outcome in 9 studies, and did not make any difference in 6 studies. There is no doubt that early mobilization after surgery is effective in preventing venous thromboembolic complications. However, in 2016 a systematic review failed to show any influence of early mobilization after abdominal and thoracic surgery on length of stay, postoperative ileus, functional tests, or degree of patient activity [59]. Regardless of the lack of high-level evidence a strong recommendation was made by the international consensus group to mobilize patients as soon as possible after surgery. 

#### Thromboembolic prophylaxis

Patients undergoing colorectal surgery are at high risk for postoperative venous thromboembolism. Therefore, the German S3 Guideline [60] recommends low-molecular heparin as medical prophylaxis. Patients can also be treated with medical compression stockings. The duration of medical prophylaxis should be 7 d at least and patients undergoing surgery for cancer should undergo prolonged medical prophylaxis for 4 weeks. If epidural or spinal analgesia is considered, the time interval between the last heparin injection and regional anesthesia should be 12 hours (in patients with regular renal function) and can be as long as 24–30 h in patients with a creatinine clearance <30 ml/min. 

All the postoperative elements of OPM are shown in Table 5 (Tab. 5).

### Adherence to protocol and outcome

During the last 25 years, the number of elements for OPM has increased substantially. For example, the current OPM recommendations for elective colorectal surgery described 24 elements that should be applied to every patient [3]. Recent studies show that simple formulation and training with a standard operating procedure only leads to the implementation of about 45–50% of all required measures [61], [62]. Among other studies, a Spanish multicenter trial [63] revealed that patients with high adherence to the OPM protocol (>77% of all elements adhered to) the rate of moderate to severe complications was only 16%, whereas 35% of patients with poor adherence (<54%) experienced moderate to severe adverse events. Oral food was tolerated after 7 hours compared to 24 hours, mobilization was completed after 24 hours compared to 48 hours and hospital stay was 5 d compared to 8 d. Comparable correlations between the degree of adherence to the OPM protocols and postoperative outcomes have also been demonstrated for gynecological [64] and orthopedic surgery [65]. To achieve high adherence rates to the OPM protocol, two prerequisites are required:

1. Monitoring of the treatment pathway and control of adherence, including the timely correction of deviations from the OPM protocol by specialized staff (so-called OPM assistants or OPM nurses) [66], [67]; and
2. Continuous feedback of the clinical implementation of the OPM protocol through an IT-supported audit system [3]. OPM assistants enter relevant data into this audit system and immediately receive standardized reports including adherence to individual elements of the OPM protocol.

### Clinical results of OPM in visceral surgery

The effects of OPM have been investigated in numerous studies. OPM reduces the frequency of postoperative complications. The length of hospital stay as a surrogate parameter for the speed of postoperative recovery is shortened by 2–3 d while there is no difference in readmission rate after OPM compared to traditional perioperative treatment. 

#### Colorectal resections

OPM reduces the complication rate from 29.1% to 20.6% when compared to traditional perioperative treatment [68]. If only laparoscopic surgery was investigated, morbidity decreased from 27.0% to 17.8% with OPM [69]. Older patients benefited particularly, with complication rates decreasing from 54.2% to 25.9% in RCTs [70]. Length of hospital stay fell by –2.62 (–3.22; –2.02) d for all patients [68] and by –2.0 (–2.5; –1.5) d for laparoscopic operations [69]. The time to first bowel movement was –32.9 (–45.5; 20.5) hours shorter after laparoscopic surgery [69]. The rate of surgical site infections was not different for all colorectal resections at 3.5% (OPM) versus 4.8% (traditional). This is consistent with meta-analyses of randomized controlled trials (RCTs) in which only the rate of non-surgical complications was reduced by OPM from 7.5 to 3.0% [71]. Patients treated with OPM had a 59% lower risk of pulmonary complications and a 49% lower risk of cardiovascular complications than traditionally treated patients.

#### Esophageal resections

In meta-analyses of RCTs [72], an odds ratio of 0.48 (0.27; 0.86) was calculated for the risk of postoperative complications with OPM compared to traditional therapy. The risk of pulmonary complications was particularly reduced with an odds ratio of 0.37 (0.18; 0.74). In addition, a lower incidence of anastomotic insufficiencies (odds ratio: 0.27 [0.07; 0.96]) was observed under OPM. Length of stay decreased by –2.89 (–3.39; –2.39) d with OPM.

#### Gastric resections

A meta-analysis of RCT [73] did not find a significant reduction of morbidity by OPM compared to traditional therapy (18.2% vs. 21.7%). The rate of pulmonary complications under OPM was 3.4% but 7.2% under traditional treatment. Length of stay was reduced by –1.8 (–2.2; –1.4) d, but the readmission rate was slightly higher after OPM (4.5%) than in patients treated traditionally (1.7%). 

#### Liver resections

The meta-analysis of RCTs [74] found morbidity reduced from 42.6% to 24.5% with OPM. Reoperation and readmission rates were not associated with the type of perioperative treatment. Again, length of stay decreased by –3.2 (–4.0; –2.4) d under OPM. There was no effect on mortality.

#### Pancreatic resections

Postoperative morbidity was not associated with the type of perioperative treatment (53.1% versus 63.1%) [75]. Pancreatic fistulae, reinterventions or reoperations, readmission rates, and mortality were also not different. However, delayed gastric emptying was about twice as often with traditional treatment (32.7%) than with OPM (16.7%). Furthermore, the length of hospital stay was –3.7 (–4.8; –2.6) d shorter when OPM was utilized.

#### Bariatric surgery

In these operations, OPM did not result in different complication rates (11.8% versus 10.9%) [76]. Rates of major morbidity (3.9% versus 2.6%) and anastomotic leakage (1.5% versus 1.6%) were also not different. However, postoperative nausea and vomiting were observed more frequently with traditional therapy (13.5% of all patients) than with OPM (6.4%). Readmission rates were comparable (4.5% versus 4.3%). Hospital length of stay was reduced by –0.5 (–0.92; –0.10) d with OPM in these patients who were often treated as short-stay inpatients. 

#### Abdominal wall reconstructions

Complex abdominal wall reconstructions under OPM led to a reduction in hospital length of stay of –0.89 (–1.70; –0.07) d with comparable recurrence (19.0% versus 18.1%) and readmission rates (12.4% versus 12.1%) in non-randomized studies [77]. 

## Conclusion

Almost 3 decades ago, clinical results of an OPM protocol were published for the first time in a small series of elective colorectal resections. Since then, OPM has spread not only to other abdominal operations performed by surgeons, gynecologists, or urologists but also to thoracic, vascular, orthopedic surgery. OPM has become the method of the first choice for these elective procedures and starts to be tested in emergency surgery too. However, the development of OPM pathways has not yet been completed and the limits of this multimodal therapy cannot be definitively defined. Drug interventions to specifically manipulate the postoperative neuroendocrine stress response, improved analgesia procedures, even more, effective methods to combat PONV and gastrointestinal atony could further accelerate the recovery process of our patients. 

Regular discharge of patients with elective colorectal resections as early as 2 d after surgery seemed implausible to many surgeons in 1995 [78]. In 2019, French surgeons reported that with OPM, they were able to perform about a third of their laparoscopic colorectal resections as outpatient procedures [79]! Whether such short postoperative stays are in the best interest of the patients may be debatable and must certainly be discussed critically. However, they show the potential of OPM. In Germany, the application of OPM in the clinical routine is rarely seen, so structured implementation of OPM is the main task in our country. Neither the shortage of nursing personnel nor the German reimbursement system stands against the nationwide adaptation of OPM protocols. On the contrary, the nursing workload is decreased by OPM [67], and decreased length of stay is a strong financial argument to implement OPM. 

In conclusion, optimized perioperative management will replace traditional perioperative treatment because it’s rare capability to improve quality, help the patient, relieve the nurses, and improve the financial situation of a hospital. 

## Notes

### Competing interests

The author declares that he has no competing interests.

## Figures and Tables

**Table 1:**
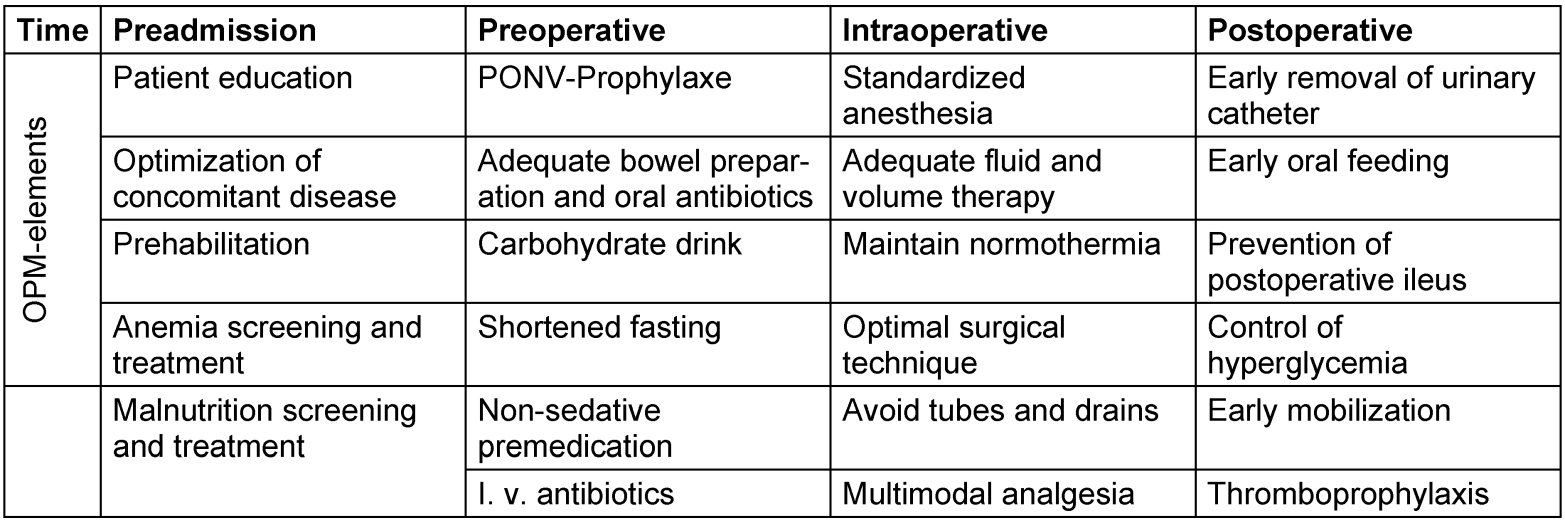
Protocol for OPM in elective colorectal surgery (adapted from [3])

**Table 2:**
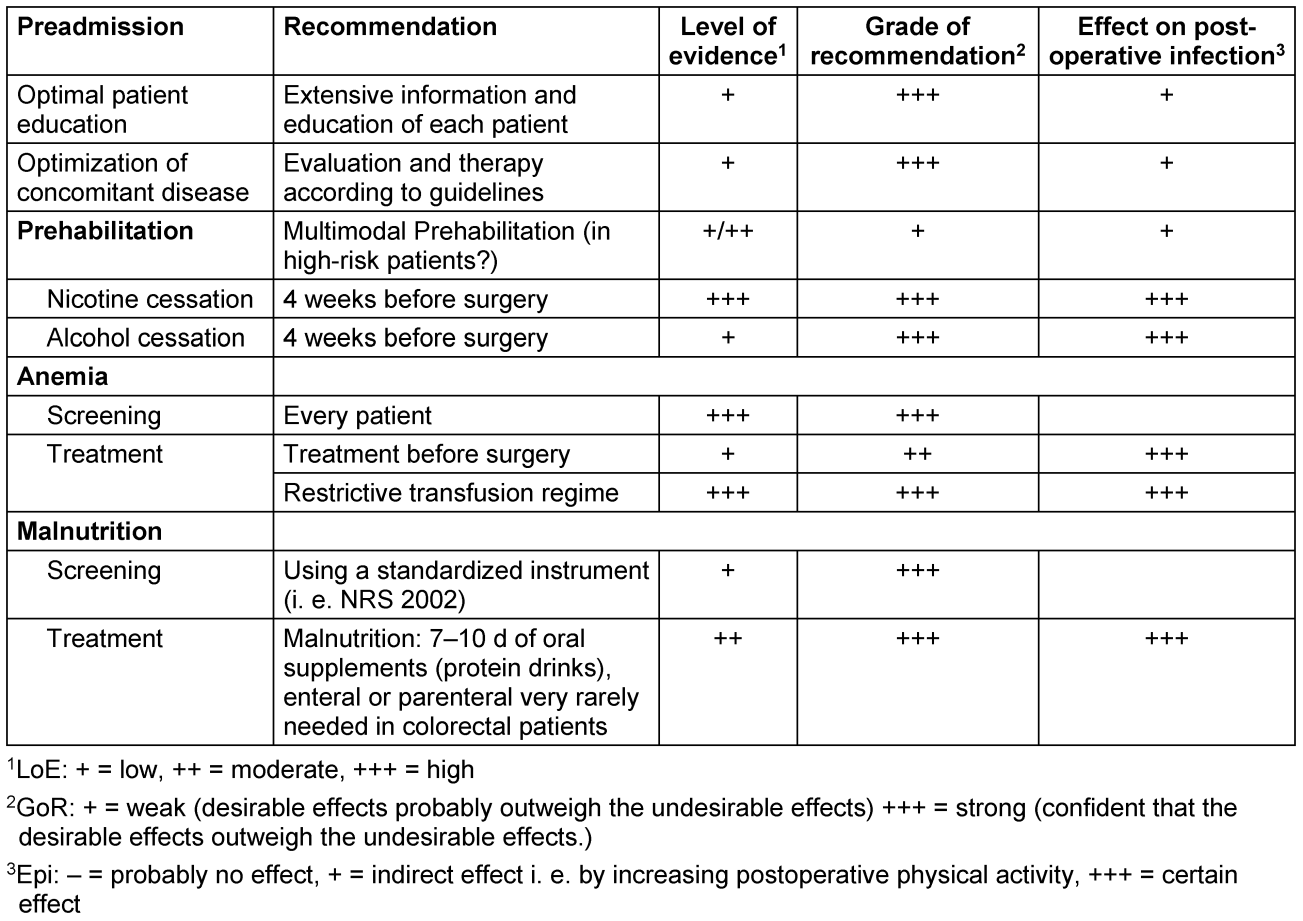
OPM elements during the preadmission period, level of evidence, grade of recommendation (according to [3]) and effect on postoperative infections.

**Table 3:**
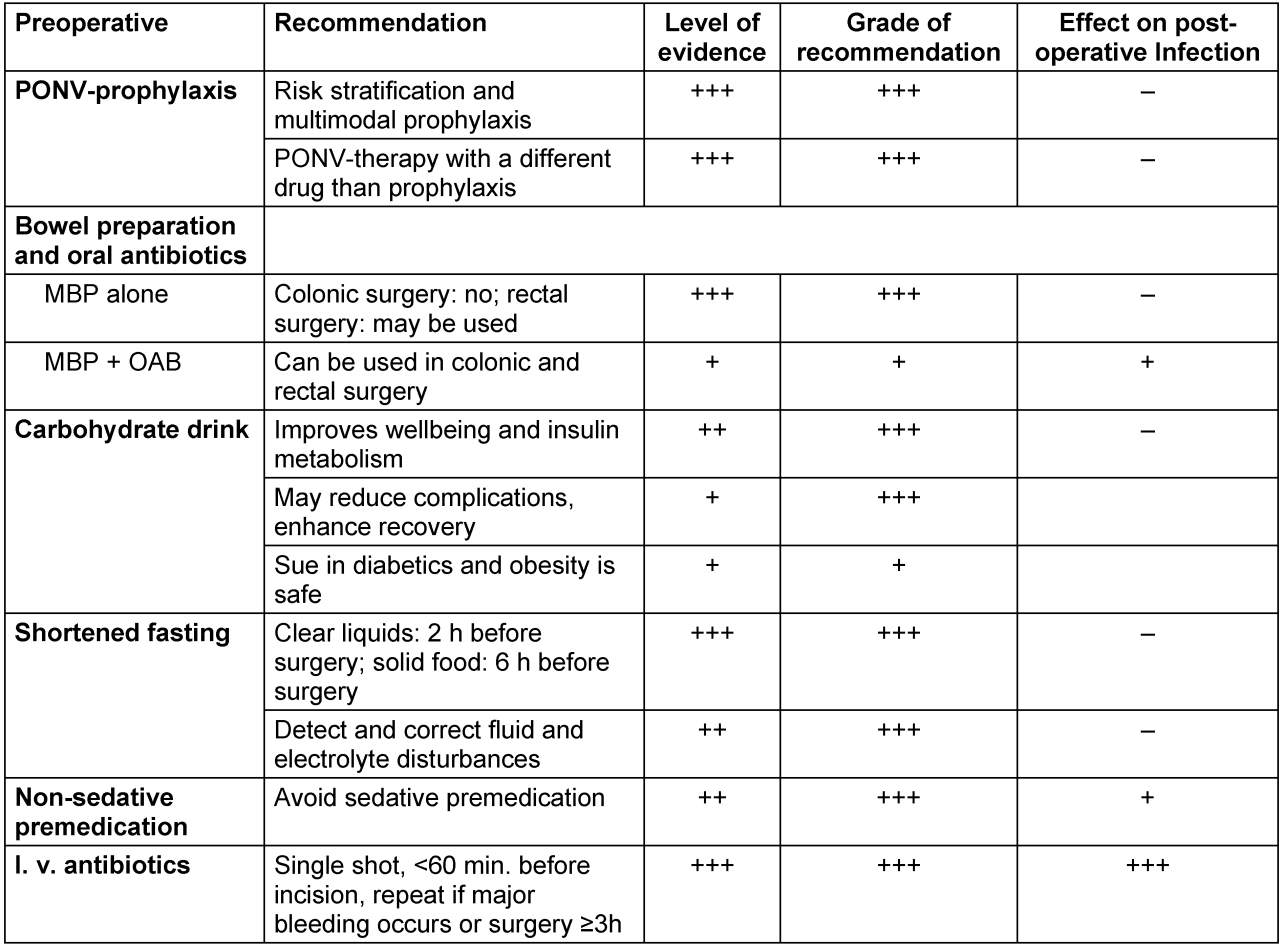
OPM elements during the preoperative period, level of evidence, grade of recommendation (according to [3]) and effect on postoperative infections.

**Table 4:**
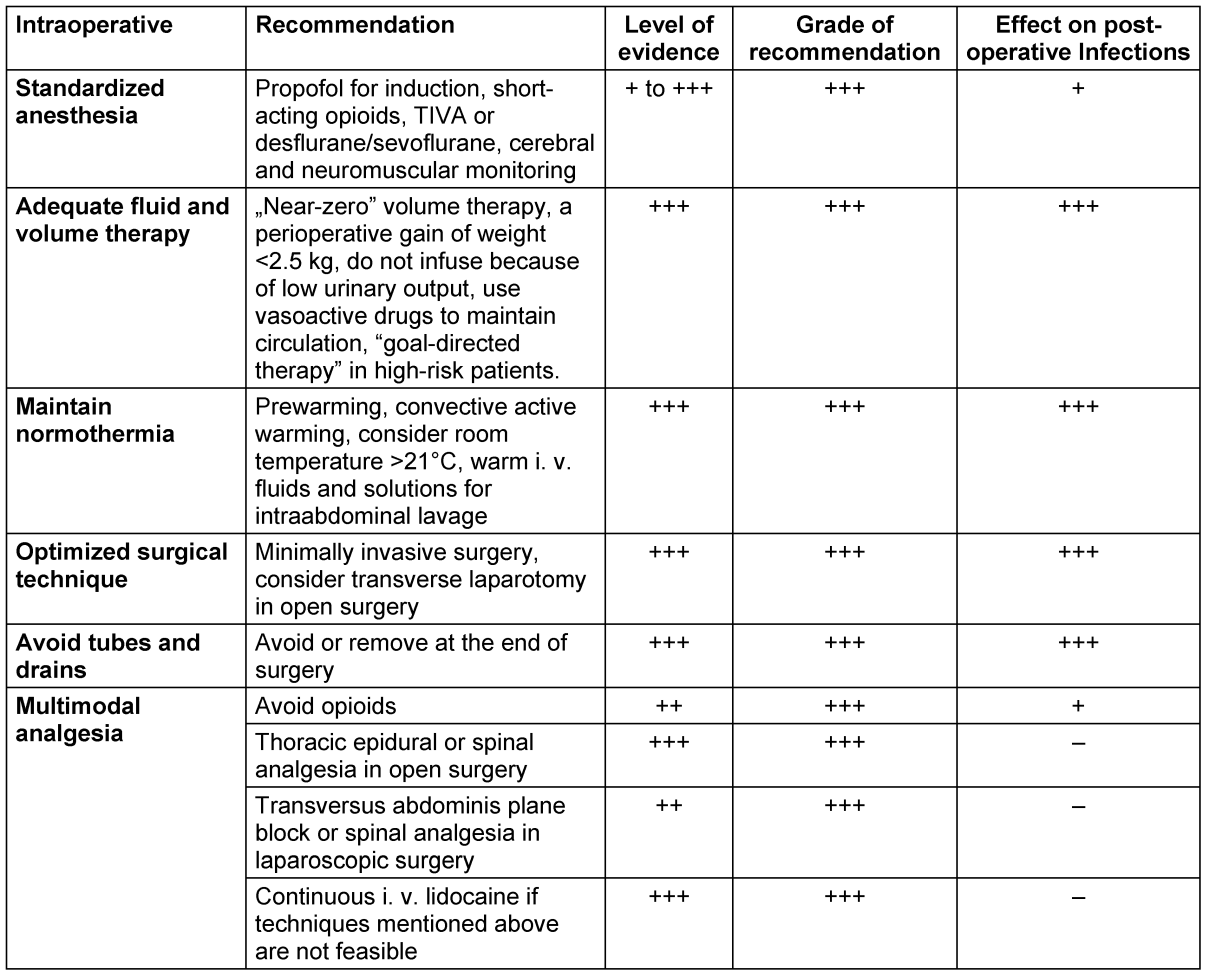
OPM elements during the intraoperative period, level of evidence, grade of recommendation (according to [3]) and effect on postoperative infections.

**Table 5:**
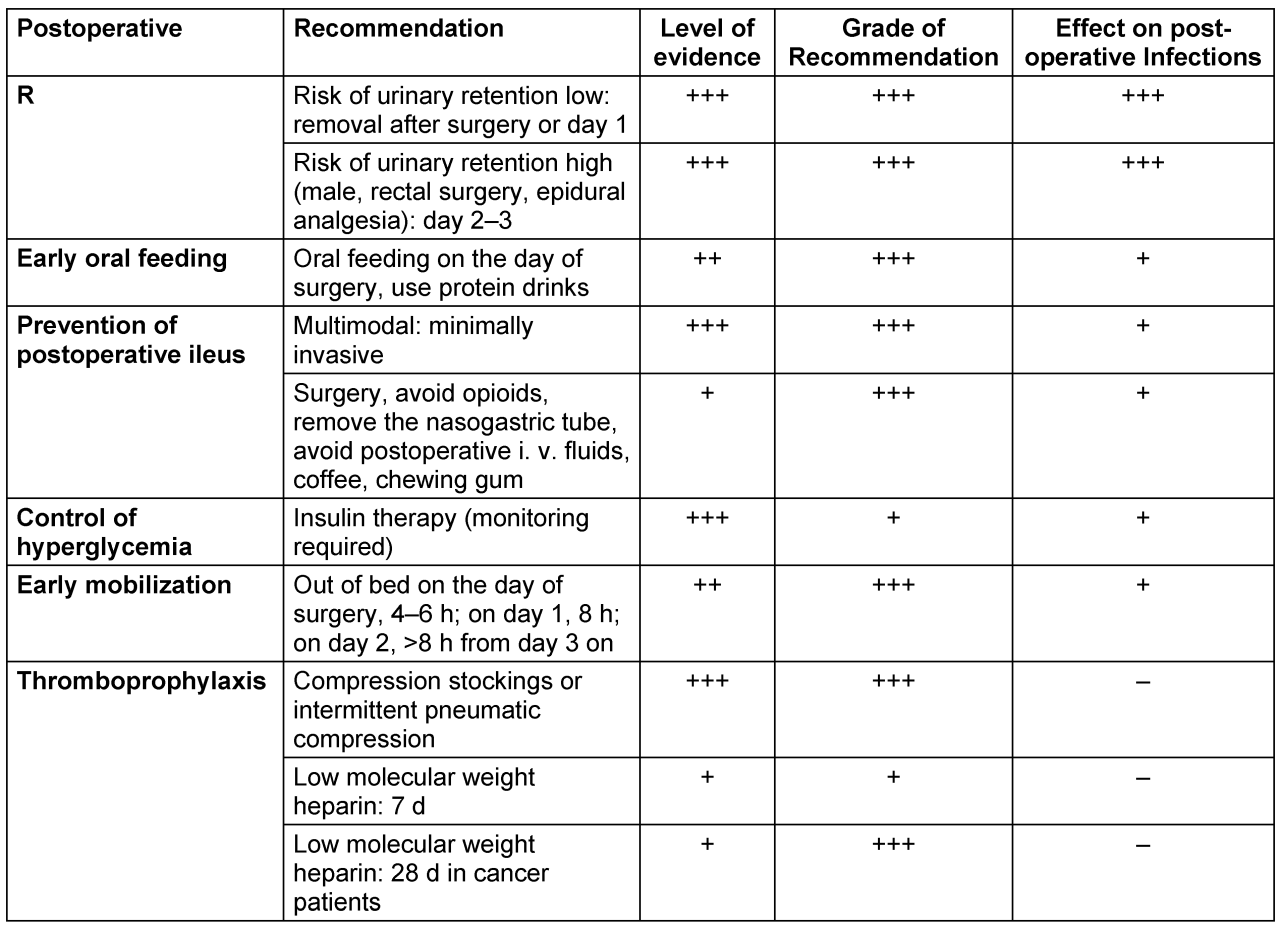
OPM elements during the postoperative period, level of evidence, grade of recommendation (according to [3]), and effect on postoperative infections.
